# Pandemic Pregnancy Experiences and Risk Mitigation Behaviors: COVID-19 Vaccination Uptake in Canada

**DOI:** 10.3390/ijerph22030425

**Published:** 2025-03-14

**Authors:** Sigourney Shaw-Churchill, Karen P. Phillips

**Affiliations:** Interdisciplinary School of Health Sciences, University of Ottawa, Ottawa, ON K1N 6N5, Canada

**Keywords:** COVID-19 pandemic, pregnancy, COVID-19 vaccines, vaccine hesitancy, prenatal care, antenatal care

## Abstract

Background: Pregnant people in Canada during the pandemic faced complex decision-making related to COVID-19 exposure risks and the safety of mitigation measures, including vaccines. To help inform future infectious disease–health promotion, we assessed pandemic pregnancy experiences and COVID-19 risk mitigation strategies. Methods: Respondents, pregnant at any time after January 2020 in Canada, completed an online, cross-sectional, descriptive survey from September 2021 to February 2022. Logistic regression was used to identify predictive factors associated with COVID-19 vaccine uptake and history of infection. Results: A purposive sample of predominantly non-racialized, high socioeconomic status women (*n* = 564), 58.2% primigravid during the pandemic, reported high COVID-19 vaccine uptake (87.4%). Educational attainment beyond high school predicted COVID-19 vaccination (college AOR: 2.72, CI: 1.24–5.94, *p <* 0.001; university AOR 4.01, CI: 1.91–8.40, *p* < 0.001; post-graduate university AOR: 7.31, CI: 2.84–18.81, *p <* 0.001). Immigrant status reduced the likelihood of COVID-19 vaccination (AOR: 0.20; CI: 0.09–0.49, *p <* 0.001). Racialized participants were 2.78-fold more likely to report infection (CI:1.19–6.50, *p =* 0.018). Conclusions: COVID-19 vaccination uptake was very high; however, vaccine hesitancy was evident among immigrants, with racialized participants more likely to report a history of COVID-19 infection. Tailored public health messaging using a health equity lens may yield more robust vaccine uptake for future infectious respiratory disease outbreaks.

## 1. Introduction

The emergence of COVID-19 in Canada in 2020 was accompanied by significant societal changes, including regional lockdowns, school closures, and transitions to telework [1]. Public health measures, such as masking, handwashing, social distancing and limits to social contacts, were broadly targeted to the general population [1]. The closure of the United States (USA)–Canada border and regional travel restrictions, including quarantine of the northern territories, curfews in Quebec and the formation of the Atlantic Bubble—comprising New Brunswick, Nova Scotia, Prince Edward Island and Newfoundland and Labrador—were among pandemic travel restrictions [1,2]. COVID-19 vaccines were introduced to the general public in 2021, culminating in about 80% of eligible Canadians vaccinated by the end of 2021 [3], one of the highest national rates of two-dose vaccine coverage [2]. However, despite the early promise of vaccines, COVID-19 variants emerged with Delta in the fall of 2021, followed by Omicron in winter 2022, which further increased rates of infection [3]. Public acceptance of COVID-19 risk mitigation measures was initially strong, with a collective attitude of perseverance, but waned as COVID-19 infection rates continued to increase despite widespread vaccine uptake and fueled by social media-propagated misinformation [3].

For high-risk and special populations, including people who were pregnant or new parents, the effects of the global pandemic were particularly stressful, given the uncertain and constantly evolving information relevant to pregnancy [4]. In Canada, about 355,000 live births occur each year, with most Canadians giving birth in hospitals attended by obstetricians [5]. More Canadians gave birth outside of hospitals, a 2.4% increase in 2021, possibly due to fear of hospital-based COVID-19 infections that occurred in 2021 [6]. During the pandemic, live birth rates in Canada, excluding Yukon, fluctuated with a 3.3% decline between 2019 and 2020, a 2.6% increase in 2021, followed by an apparent 5% decrease in 2022 [6,7]. The pandemic’s effects on family planning decisions, inflation, along with a 45% decrease in the admittance of immigrants to Canada in 2020, likely explain some of these patterns [6]. Initially, there were significant information gaps about the severity of COVID-19 during pregnancy and possible transmission risks to fetuses and newborns, though breastfeeding continued to be promoted [8]. Despite the well-established morbidity and mortality for pregnant people [9,10] infected with seasonal influenza, H1N1, and COVID-19’s predecessor, SARS-CoV-1, both Canada [11] and the USA [12] initially recommended against routine COVID-19 vaccination during pregnancy. Canadian public health recommendations for pregnant people remained similar to that of the general population throughout the pandemic—practice social distancing, wear a mask in public places, wash hands frequently, and stay home [13]. As global clinical experience with COVID-19 increased, it became apparent that pregnancy was associated with more severe illness and associated complications, with Canada [13] and the USA [14] ultimately identifying pregnancy as a priority condition for COVID-19 vaccination.

The disproportionate impacts of pandemics on high-risk populations, including pregnant people, are further magnified by health inequities due to racism, colonialism, poverty, and citizenship [15]. Even before the pandemic, sociodemographic characteristics influenced seasonal influenza vaccination uptake in Canada [16] and the United States [17]. Future pandemics will once again require adherence to public health measures, including vaccination. We therefore investigated pandemic pregnancy experiences, behaviors, and strategies used to mitigate COVID-19 risks in Canada.

## 2. Methods

### 2.1. Sample

Participants aged 18–45 years, who were pregnant at any time after January 2020 and who accessed pregnancy care in Canada, were invited to participate in an online survey (SurveyMonkey^TM^, San Mateo, CA, USA). Participants were recruited via social media channels and advertisements, community groups, and networks from 1 September 2021 to 1 February 2022. The non-random sample was purposive, aiming to provide a descriptive portrait of pregnancy experiences during the pandemic in Canada. We determined a sample size of 384 based on annual live birth rates in Canada [5,6]. To ensure respondents had relevant experiences with Canadian public health and perinatal healthcare systems, recent immigrants to Canada (<5 years) and Canadians living abroad or with pregnancy experiences outside of Canada, along with respondents unable to understand English, were excluded from participation.

### 2.2. Survey

The Pandemic Pregnancy Experiences Study, a cross-sectional, descriptive survey, was designed to capture reproductive decision-making behaviors, pregnancy care experiences, and psychosocial stress/social supports during COVID-19. For this analysis, an excerpt of the broader survey explored (i) prenatal experiences (prenatal care region COVID-19 rates, prenatal healthcare provider (HCP) type, gravidity), (ii) pandemic risk mitigation behaviors/characteristics (household size, characterization of unmasked social contacts, non-essential travel, COVID-19 vaccinations, COVID-19 infection history), (iii) employment (telework, essential worker status), and (iv) demographic information. Survey questions were formatted as Likert scales, multiple responses, and yes/no responses.

### 2.3. Analysis

Survey responses were exported to Excel^®^ (Microsoft Office, Version 2304; Microsoft, Redmond, Washington, DC, USA), incomplete and missing data removed, followed by analysis in SPSS (IBM Statistics 28.0.1.1(14)). We received a total of 1062 consented responses to the full survey; 498 responses were eliminated from this sample as they were not relevant to this analysis (secondary survey objective) or did not provide demographic, pregnancy details, and household size information (surveys considered incomplete). The sociodemographic characteristics of the sample are presented as descriptive statistics. Gravidity was denoted as ‘primigravid’ for first pregnancies during COVID-19 and ‘multigravid’ for participants with reported pregnancies both before 1 January 2020 and during the pandemic. Predictive factors related to two main outcome variables, (i) COVID-19 vaccination and (ii) history of COVID-19 infection, were evaluated. Sociodemographic characteristics, gravidity, employment, local COVID-19 rates, and domestic excursions were assessed as potential predictive variables by bivariate analyses (Pearson Chi-Square (χ^2^) for nominal/dichotomous variables, rank-biserial correlation for ordinal variables, and point-biserial correlation for continuous variables). Statistically significant variables (*p* < 0.05) were retained for logistic regression models, with extreme outliers (>±3 standard deviations) removed for household size. The outcome variables, COVID-19 vaccination and history of COVID-19 infection, were each dichotomized (yes/no) based on participant self-report. Note that we did not collect information on the number of vaccine doses received or vaccine brands. Potential predictive variables were included in separate binomial logistic regressions to determine the probability of either COVID-19 vaccination or infection. Predictive variables for each outcome were identified based on crude odds ratio (COR), adjusted odds ratio (AOR), and corresponding confidence intervals (CI).

### 2.4. Ethics

Survey respondents were provided with details about the study, the benefits and risks associated with participation, and could enter a draw for a CAD 50 Amazon gift card to be compensated for their time. The Research Ethics Board at the University of Ottawa approved this study (File #H-05-21-6902). All respondents indicated informed consent prior to their participation in the survey.

## 3. Results

### 3.1. Sample

A total of 564 women who were pregnant during the pandemic comprised our purposive sample, exceeding our sample size calculation of 384 (Table 1). The sample was predominantly non-racialized, with an average age of 32.2 years, and relatively high socioeconomic status (SES), as indicated by university/postgraduate university degree completion (*n* = 371; 65.8%), and annual household incomes in excess of CAD 100,000 (*n* = 342; 60.6%). Slightly more than half of the participants were primigravid during the pandemic (58.2%) and received prenatal care in Ontario (50%). Pregnancy loss was reported by 14 primigravid and 9 multigravid respondents, and the experience of multiple pregnancies during the pandemic was reported by 11 respondents.

### 3.2. COVID-19 Experiences

Most participants received prenatal care in a region perceived to have moderate to high COVID-19 rates (53.7%; Table 2). Participant household size during the pandemic was 2.86 ± 1.09 members, with 84.4% of participants (*n* = 476) having regular close, unmasked social interactions beyond their immediate household members, primarily with family and friends. Feeling socially isolated was reported by about half of the sample (often/always: *n* = 288; 51.1%). Most of the sample limited non-essential trips outside the home (often/always: *n* = 389; 69%). The majority of participants reported travel outside of their home region within the past month (*n* = 354; 62.8%), mainly to visit family and access prenatal services. At the time of the survey, most participants were vaccinated (*n* = 494, 87.4%), with over half of the sample vaccinated while pregnant (*n* = 294; 52%). The history of COVID-19 infection across the sample was very low (9.2%). Among respondents with a history of COVID-19 infection, two-thirds contracted COVID-19 while pregnant (*n* = 35; 67.3%).

### 3.3. Predictors of COVID-19 Vaccination

Of the 15 factors evaluated as potential predictors of COVID-19 vaccination, 4 were statistically significant (Table 3): (1) immigrant status (yes/no; χ^2^(1) = 10.90, *p =* 0.004), (2) education (some high school/high school, college, university, postgraduate degree; U = 12,020.0, Z = −3.84, *p <* 0.001), (3) household income (see Table 1 for income categories) U = 10770.5; Z = −2.69, *p =* 0.007), and (4) gravidity (primigravid, multigravid; χ^2^(1) = 4.38, *p =* 0.047). Household income was not retained for further analysis, as this variable reflected combined incomes of all household members, rather than individual incomes, and further, income was positively correlated to educational attainment (Spearman’s rank correlation (536) = 0.41, *p <* 0.001). A logistic regression was performed to ascertain the effects of immigrant status, gravidity, and education on the likelihood of COVID-19 vaccination (Table 4). The logistic regression model was statistically significant, χ^2^(5) = 32.17, *p <* 0.001. The model explained 10.8% (Nagelkerke *R^2^*) of the variance in vaccination and correctly classified 88.4% of cases. The Hosmer–Lemeshow Test was not significant (χ^2^(5) = 1.19, *p =* 0.95). Although primigravid status significantly increased COVID-19 vaccination in the bivariate analyses (COR: 1.72, CI: 1.03–2.88), this effect was lost after adjustment for immigrant status and education. Immigrant status reduced the likelihood of COVID-19 vaccination (AOR: 0.20; CI: 0.09–0.49, *p <* 0.001), whereas educational attainment beyond high school significantly increased the likelihood of COVID-19 vaccination (college AOR: 2.72, CI: 1.24–5.94, *p <* 0.001; university AOR: 4.01, CI: 1.91–8.40, *p <* 0.001; post-graduate university AOR: 7.31, CI: 2.84–18.81, *p <* 0.001).

### 3.4. History of COVID-19 Infection

Of 17 factors evaluated as potential predictors of history of COVID-19 infection, 4 were statistically significant (Table 5): (1) household size (*r*(559) = 0.11, *p =* 0.01), (2) racialized status (yes, no; χ^2^(1) = 5.90, *p =* 0.023), (3) immigrant status (yes/no; χ^2^(1) = 4.78, *p* = 0.042, and (4) gravidity (primigravid, multigravid; χ^2^ (1) = 5.91, *p =* 0.018). Immigrant status was positively correlated to racialized status (χ^2^(1) = 78.19, *p* < 0.001) and was, thus, not retained for the logistic regression analysis. The effects of racialized status, gravidity, and household size on the likelihood of a history of COVID-19 infection were assessed by logistic regression (Table 6). The logistic regression model was statistically significant, χ^2^(3) = 11.75, *p =* 0.008. The model explained 4.6% (Nagelkerke *R^2^*) of the variance in vaccination and correctly classified 91.1% of cases. The Hosmer–Lemeshow Test was not significant (χ^2^(5) = 6.46, *p =* 0.26). Primigravid status was negatively associated with COVID-19 infection history (COR: 0.49; CI: 0.28–0.88), with household size (COR:1.40; CI: 1.08–1.81), and racialized identity (COR: 2.71; CI: 1.18–6.24) positively correlated in the preliminary model. Only racialized identity remained significantly associated with COVID-19 infection history (AOR: 2.78; CI: 1.19–6.50) in the final model.

## 4. Discussion

Our findings provide a snapshot of pandemic pregnancy experiences at the time of our national Canadian survey in 2021—early 2022. Respondents generally limited unmasked social contacts to their extended family members and close friends, and curtailed non-essential trips outside their homes, which culminated in feelings of social isolation. Most of our Canadian sample reported moderate–high rates of COVID-19 in prenatal care regions and were vaccinated against SARS-CoV-2, but few reported a history of COVID-19 infection, perhaps due to high national vaccination uptake, mask mandates, and widespread public health restrictions [3]. In our sample, immigrant status decreased the likelihood of vaccination, and increasing educational attainment was a positive predictive factor of COVID-19 vaccination. Although household size during the pandemic, racialized status, immigrant status, and gravidity were all significantly associated with the history of COVID-19 infection, only racialized status was a predictive factor for infection.

### 4.1. COVID-19 Vaccination Uptake

Most of our survey population was vaccinated against COVID-19, related in part to widespread uptake of vaccines by the Canadian public and the introduction of mandatory policies requiring vaccination for government workers and employees from a range of sectors, including healthcare, education, and municipal agencies [3,18]. More than half of the sample was vaccinated while pregnant, generally consistent with global meta-analyses, which estimate COVID-19 vaccine acceptability during pregnancy at about 50% [19,20] and vaccine uptake by pregnant Canadians of 57.5–83.7% by mid-2022 [21,22,23]. Another Canadian study of preconception, pregnant, and lactating Canadians identified scientific evidence and promotion by maternity healthcare providers and public health officials as motivations for COVID-19 vaccine uptake [24]. The pregnancy-COVID-19 vaccine acceptability rate in our population is likely greater than 52%, given that an additional 15.8% were vaccinated while breastfeeding, and that not all participants would have been able to be vaccinated during pregnancy due to differential regional vaccine availability and slow prioritization of pregnancy as a vaccine-eligible group [12,13,23]. Further, as our sample included participants who were pregnant at any time during the pandemic, COVID-19 vaccine dosing schedules, which were dependent on vaccine type, public health region, and priority group status [2], may not have coincided with participants’ pregnancies.

Educational attainment was a major predictor of COVID-19 vaccination uptake in our pandemic pregnancy experiences sample, with the completion of college (2.7-fold), university (4-fold), and postgraduate education (7.3-fold) each increasing likelihood of vaccination compared to participants who had completed some or all of high school. SES broadly influences reproductive decision-making, with previous systemic reviews demonstrating an association between higher education, income, and COVID-19 vaccine acceptance [19,20,25], consistent with our findings. Low income and material deprivation, markers of SES marginalization, were among the factors associated with low COVID-19 vaccine uptake in pregnant Ontarians [23]. Household income, correlated to educational attainment, was associated with COVID-19 vaccination, but other SES variables, including employment status and the ability to work from home, were not related to vaccine uptake in our sample. The role of low educational attainment on health decision-making and outcomes is often confounded by other sociodemographic characteristics, including race, income, geography,; however, education does seem to relate more directly to health literacy—the capacity to comprehend health information and take appropriate actions to promote and maintain health [26].

In the context of COVID-19, higher education provides greater health literacy, which translates to enhanced ability to assess both established and emerging health information and to better understand and employ risk mitigation strategies to reduce adverse outcomes [26]. Studies of reproductive-aged and pregnant Canadians identified that vaccine uptake was related to self-reported knowledge of COVID-19 severity and pregnancy, with vaccine hesitancy attributed to safety concerns and lack of data related to vaccine efficacy [22,24]. Similarly, a systematic review of American studies concluded that COVID-19 vaccine hesitancy was related to a lack of understanding of vaccine development and approval [27]. Health information seeking is made more complicated by the extensive misinformation available on social media, with those with less education more likely to believe false, inaccurate, or incorrect COVID-19 narratives [28]. For example, social media misinformation falsely suggesting causal links between COVID-19 vaccines and infertility or miscarriage was purported to slow or prevent vaccine uptake during pregnancy or conception [29], consistent with concerns expressed by unvaccinated Canadians [24]. Lack of prenatal care, associated with SES deprivation, limits sources of health information and opportunities for vaccine promotion [23]. Healthcare provider discussions were positively associated with COVID-19 vaccine uptake by preconception, pregnant or lactating Canadians and identified as an influential factor in unvaccinated Canadians’ willingness to be vaccinated [24]. Healthcare providers can mitigate vaccine hesitancy by building patient confidence in the safety and benefits of vaccination, correcting misinformation, addressing patient concerns, and continuously offering vaccines, even after initial refusals [30,31].

Immigrant status was the second major predictor of COVID-19 vaccination uptake in our Canadian sample, with immigrants 80% less likely to be vaccinated compared to non-immigrants. The relatively small number of immigrant participants (*n* = 29) in our study identified as 48% racialized, 62% born to a country outside North America/Western Europe, and 41% having immigrated to Canada after the age of 20 years. Higher rates of university/post-graduate degree completion were reported by immigrant participants (79.3%) compared to domestic participants (65%), suggesting that educational attainment was not related to vaccine uptake in this subgroup. Our recruitment criteria promoted the survey to respondents who had lived in Canada for at least 5 years to ensure some degree of acculturation and social integration [32], such that more recent immigrants may exhibit even higher rates of COVID-19 vaccine hesitancy while pregnant. Although access to obstetrics care is generally similar among immigrants and non-immigrants in Canada, postpartum follow-up [33] and access to general practitioners [34] may limit health promotion opportunities. A large Canadian study of non-pregnant Canadian immigrants reported a COVID-19 vaccine hesitancy prevalence of 21.5%, despite their increased exposure risk due to employment-related public interactions and multigenerational households [34]. Vaccine hesitancy among racialized Canadian immigrants is suggested to be related to fear of stigma and xenophobia [34], consistent with the historically poor pregnancy experiences reported by racialized and Indigenous people of Canada [35] and the United States [36] during childbirth. Fostering more inclusive, culturally safe provider–patient interactions in pregnancy care [35,36] is essential to mitigate vaccine hesitancy during pregnancy [31].

### 4.2. COVID-19 Infection History

A small proportion of our sample (9.2%) reported a history of COVID-19 infection but no hospitalization, despite the emergence of several waves in 2021, including Delta and the highly transmissible Omicron variants [3]. This small subsample mainly included participants who were pregnant/breastfeeding (84.6%) at the time of the COVID-19 infection. Estimates of COVID-19 prevalence range considerably, with a 4.5% seroprevalence among Ottawa, Canada, obstetric patients in 2020 [37], 5% prevalence among Montreal, Canada, obstetric patients in 2020, and a 5.6% prevalence of positive COVID-19 tests reported by a sample of pregnant Canadians in 2021–2022 [22]. A global systematic review/meta-analysis reported significantly higher prevalence rates of COVID-19 (>15%) from American studies, with an overall rate of hospital-based COVID-19 diagnosis of 9% for pregnant and recently pregnant women [38,39]. The relatively low COVID-19 prevalence rates in our sample may be explained by high vaccination rates but also widespread masking, regional restrictions, and lockdowns [3]. The majority of the sample (69%) reported often/always limiting non-essential trips outside the home, undoubtedly contributing to over half of the sample reporting often/always feeling socially isolated. Social isolation, both self-imposed and due to public health measures, has been described for pregnant people during the pandemic [40].

In our sample, large household size, racialized status, multigravid and immigrant status were characteristics associated with history of COVID-19 infection, with only racialized status (2.78-fold increase in COVID-19 infection history) being a positive predictor for history of infection, consistent with findings from a global meta-analysis [38,39] and a large historic United Kingdom seroprevalence study of antenatal serum samples [41]. Mean household size (1.78) and essential worker status proportion (75%) were both slightly higher among racialized participants compared to non-racialized participants (1.61, 65%); however, vaccination rates were similar ). A greater proportion of racialized participants identified as immigrants to Canada, which, as discussed, was also associated with reduced COVID-19 vaccination uptake. In addition to racialized ethnicities, other risk characteristics associated with COVID-19 infection during pregnancy identified in the literature include high body mass index [38,39], measures of multiple deprivation, and age younger than 30 years [41]. The identification of individual risk characteristics associated with COVID-19 exposure is useful to inform tailored risk estimates for the purposes of health education and public health messaging in future community-level health emergencies.

### 4.3. Limitations

This study captures COVID-19 mitigation measures in a sample of women who experienced pregnancy during the pandemic in Canada. Although the findings will have important implications for the development of targeted public health messaging and perinatal healthcare, we identify several limitations of our study. First, the overall high levels of education and income of the survey respondents do not reflect the SES of most Canadians, as many of our respondents able to mitigate pandemic exposures through telework and economic privilege. We also acknowledge that despite our attempts to recruit a diverse sample, the majority of respondents were Caucasian and identified as cis-gendered women. Our decision to recruit participants who lived in Canada for at least 5 years, together with our limited recruitment efforts targeting immigrant communities, precluded a meaningful examination of the COVID-19 risk reduction strategies by this heterogeneous population. COVID-19 rates and mitigation measures varied both over time and across regions throughout the pandemic, with our survey’s geographical diversity—including rural, remote, and urban experiences—limited in part by the use of an English-only survey instrument. We did not make all survey questions mandatory, out of consideration for sensitive experiences with pregnancy loss or stillbirth, such that our sample size was not consistent across study variables. We did not collect data on the number of COVID-19 vaccinations received, nor did we inquire whether respondents had multiple COVID-19 infections. Finally, we did not consider in the original design of our survey that due to the duration of the pandemic, some respondents had their first pregnancy experience during the pandemic, but then continued to have subsequent pregnancies, including pregnancy losses, such that we were not able to capture the full experiences of these respondents.

## 5. Conclusions

Overall, pandemic pregnancy experiences in Canada included receiving prenatal care in regions with moderate–high COVID-19 rates and limitations to non-essential and social activities to reduce COVID-19 exposures. Our findings indicate that even within the context of widespread reproductive misinformation, COVID-19 vaccination uptake was very high, with most of our sample vaccinated during pregnancy/breastfeeding. Disparities were evident; however, immigrant respondents were less likely to be vaccinated, and racialized respondents were more likely to report a history of COVID-19 infection. Targeted health messaging to immigrant and racialized communities using a health equity lens may serve to address future gaps in vaccine uptake. As pregnancy is an established risk factor for COVID-19 and other infectious respiratory disease complications, it is essential to better understand and mitigate vaccine hesitancy among equity-seeking populations.

## Figures and Tables

**Table 1:** Demographics.

Characteristic	*n*	%
Age (mean, SD)	32.2 ± 4.17	
Indigenous	34	6.0
Racialized	40	7.1
Immigrant	29	5.1
**Gravidity ^1^**		
Primigravid	328	58.2
Multigravid	236	41.8
**Education ^2^**		
Some high school/high school	55	9.8
College	138	24.5
University	234	41.5
Post-graduate	137	24.3
**Income ^3^**		
<$40,000	32	5.7
$40,000–69,999	35	6.2
$70,000–99,999	129	22.9
$100,000–149,999	183	32.5
>$150,000	159	28.2
Prefer not to answer	25	4.4
**Location of Prenatal Care**		
British Columbia	24	4.3
Alberta	40	7.1
Saskatchewan	28	5.0
Manitoba	41	7.3
Ontario	282	50.0
Quebec	34	6.0
Atlantic Bubble ^4^	102	18.1
The North ^5^	10	1.8

^1^ First pregnancy experience during pandemic (primigravid), pregnancy experiences prior to pandemic (multigravid). ^2^ Historically, Canadian ‘colleges’ have emphasized skills training, technical trades and applied learning, whereas ‘universities’ are degree-granting institutions, with many offering graduate and professional programs. ^3^ Income categories reported in Canadian dollars. ^4^ The ‘Atlantic Bubble’ was a term coined during the pandemic to reflect the pandemic travel restrictions for the Canadian Atlantic provinces: New Brunswick, Nova Scotia, Prince Edward Island and Newfoundland and Labrador. ^5^ Similarly, stringent travel restrictions protected the Northern territories (Northwest Territory, Nunvavut and Yukon) during the pandemic. SD = standard deviation.

**Table 2:** COVID-19 Experiences.

Characteristic	*n*	%	Characteristic	*n*	%
**COVID-19 Rates in Prenatal Care Region ^1^**			**COVID-19 Vaccination**	**494**	**87.4**
High	145	25.7	Yes- while trying to conceive	60	10.6
Moderate	158	28	Yes—when pregnant	294	52
Low–No COVID-19	78	13.8	Yes—when breastfeeding	89	15.8
**Household size (mean, SD)**	2.86 ± 1.09		Yes (not pregnant, not breastfeeding)	51	9
**Regular close, social, unmasked contact**			Not vaccinated	67	11.9
Limited to household members	86	15.2	**History of COVID-19 infection**	52	9.2
Non-household contacts	476	84.4	Infected when trying to conceive	3	0.5
**Non-household contacts included (*n* = 476, check all):**			Infected when pregnant	35	6.2
Extended family members	346	72.7	Infected when breastfeeding	9	1.6
Close friends	334	70.2	Infected, not pregnant, not breastfeeding	5	0.9
Work colleagues/customers/clients	119	25.0	No history COVID-19 infection	512	90.6
Daycare/childcare providers	55	11.6	**Travel outside of home city/town in past month**		
Religious community	18	3.8	No travel	205	36.3
Home services (e.g., cleaning, repair)	16	3.4	Travel	354	62.8
**Felt socially isolated**			**Purpose of travel (*n* = 354; check all):**		
Always/often	288	51.1	Visiting extended family	233	65.8
Sometimes	227	40.2	Prenatal/maternity healthcare services	143	40.4
Rarely/never	49	8.7	Visiting friends	81	22.9
**Limited non-essential trips outside home**			Work	66	18.6
Always	158	28.0	Non-reproductive healthcare services	59	16.7
Often	231	41.0	Social event	58	16.4
Sometimes	141	25.0	Vacation/activities	18	5.1
Rarely–Not at All	34	6.0	Religious/spiritual event	17	4.8
			Education	8	2.3
			Shopping/supplies	7	1.9

^1^ City/town where respondent received most of their prenatal care was characterized by respondents as High (a COVID-19 hot-spot; frequent or lengthy local lockdowns, regular outbreaks and restaurants/gyms mostly closed), Moderate (moderate COVID-19 infection rates, occasional local lockdowns, some outbreaks and restaurants/gyms sometimes closed.), Low (low COVID-19 infection rate, lockdown only because of province, infrequent outbreaks, restaurants/gyms mostly open), and No COVID-19 (What pandemic? Life has been pretty normal, no lockdowns, no outbreaks, all businesses open). SD = standard deviation.

**Table 3:** Variables associated with COVID-19 vaccination.

Variables	Vacc.		Unvacc.		Variables	Vacc.	%	Unvacc.	%
**Sociodemographic Characteristics (*n* = 561)**					**Gravidity (*n* = 561) χ^2^(1) = 4.34, *p =* 0.047**				
Age (mean, SD)	32.4 ± 4.1		31.61 ± 4.66		Primigravid	295	90.5	31	9.5
Household size (mean, SD)	2.84 ± 1.06		3.09 ± 1.30		Multigravid	199	84.7	36	15.3
	*n*	%	*n*	%	**Prenatal HCP (*n* = 379)**				
Racialized (*n* = 40)	35	87.5	5	12.5	OB/GYN	176	89.8	20	10.2
Indigenous (*n* = 34)	28	82.4	6	17.6	GP	81	84.4	15	15.6
Immigrant (*n* = 31) Χ^2^(1) = 10.90, *p =* 0.004	20	69.0	9	31.0	Midwife	65	83.3	13	16.7
**Education (*n* = 561) U = 12,020.0, Z = −3.84, *p <* 0.001**					Nurse/NP	6	85.7	1	14.3
Some high school/high school	38	70.4	16	29.6					
College	119	86.9	18	13.1	**HCP discussed vaccine (*n* = 329)**	223	86.8	34	13.2
University	208	89.3	25	10.7					
Postgraduate degree	129	94.2	8	5.8	**Employment Status (*n* = 557)**				
**Household Income (*n* = 535) U = 10770.5; Z = −2.70, *p =* 0.007**					Full-time/part-time/seasonal	307	90.3	33	9.7
<CAD 40,000	24	75.0	8	25.0	Work/maternity leave	141	86.0	23	14.0
CAD 40,000–69,999	30	85.7	5	14.3	Unemployed/leaves of absence	43	81.1	10	18.9
CAD 70,000–99,999	111	86.0	18	14.0	**Employment Characteristics**				
CAD 100,000–149,999	168	92.8	13	7.2	Essential ^1^ worker (*n* = 564 ^2^)	330	88.7	42	11.3
>CAD 150,000	145	91.8	13	8.2	Could work from home (*n* = 563 ^2^)	251	88.4	33	11.6
**COVID-19 rates ^3^ (*n* = 379)**					**Worked outside the home while pregnant (*n* = 561)**				
High	124	86.7	19	13.3	Always/often	281	88.1	38	11.9
Moderate	137	86.7	21	13.3	Sometimes/rarely	86	89.6	10	10.4
Low–no COVID-19	67	85.9	11	14.1	Never/did not work	127	87.0	19	13.0

^1^ Pandemic essential workers were generally defined by provincial/federal governments as members of employment sectors responsible for health/safety (first responders, healthcare workers), critical infrastructure operations, manufacturing, and supply chains. ^2^ Not applicable responses excluded from analysis. ^3^ COVID-19 prenatal regional infection rates as in Table 2. Relationship between respondent age, household size with vaccination history evaluated by point-biserial correlation. Mann–Whitney test used to determine the relationship between respondent education, income, and work outside home while pregnant. Pearson Chi-Square (χ^2^) test used to assess relationships between respondent race, Indigeneity, immigrant status, gravidity, employment characteristics, and HCP vaccine discussion. SD-standard deviation, HCP-healthcare provider, OB/GYN-obstetrician/gynecologist, GP-general practitioner, NP-nurse practitioner.

**Table 4:** Logistic regression analysis of variables associated with COVID-19 vaccination.

Variable	COR	95% CI	AOR	95% CI
Immigrant status (CAD ^1^)	0.27	0.12–0.62	0.20 ***	0.09–0.49
Gravidity (multigravid ^1^)	1.72	1.03–2.88	1.61	0.94–2.75
**Education** (some high school/high school ^1^) ***				
College	2.78	1.29–5.99	2.72 *	1.24–5.94
University	3.50	1.71–7.17	4.01 ***	1.91–8.40
Postgraduate	6.79	2.70–17.08	7.31 ***	2.84–18.81

^1^ Reference. COR—crude odds ratio, AOR—adjusted odds ratio, CAD—Canadian, CI—confidence interval. * *p* < 0.05; *** *p* < 0.001.

**Table 5:** Variables associated with a history of COVID-19 infection.

Variables	Infect. Hist.		No Hist.		Variables	Infect. Hist.	%	No Hist.	%
**Sociodemographic Characteristics (*n* = 564)**					**COVID-19 Rates ^3^ (*n* = 381)**				
Age (mean, SD)	32.06 ± 3.99		32.32 ± 4.19		High	19	13.1	126	86.9
Household size (mean, SD) *r*(559) = 0.109, *p* = 0.01	3.38 ± 1.48		2.81 ± 1.03		Moderate	15	9.5	143	90.5
					Low-no COVID-19	7	9.0	71	91.0
	*n*	%	*n*	%					
Racialized status (*n* = 40) χ^2^ (1) = 5.90, *p* = 0.023	8	20.0	32	80.0	**Limited non-essential trips outside home (*n* = 564)**				
Indigenous status (*n* = 34)	4	11.8	30	88.2	Always/often	35	9.0	354	91.0
Immigrant status (*n* = 29) χ^2^ (1) = 4.78, *p* = 0.042	6	20.7	23	79.3	Sometimes	12	8.5	129	91.5
**Income (*n* = 538)**					Rarely/not at all	5	14.7	29	85.3
<CAD 40,000	5	15.6	27	84.4					
CAD 40,000–69,999	4	11.4	31	88.6	**Gravidity (*n* = 564) χ^2^(1) = 5.91, *p* = 0.018**				
CAD 70,000–99,999	11	8.5	118	91.5	Primigravid	22	6.7	306	93.3
CAD 100,000–149,999	21	11.5	162	88.5	Multigravid	30	12.7	206	87.3
>CAD 150,000	10	6.3	149	93.7	**Prenatal healthcare provider (*n* = 379)**				
**Education (*n* = 564)**					OB/GYN	21	10.7	176	89.3
Some high school/high school	7	12.7	48	87.3	GP	7	7.2	90	92.8
College	15	10.9	123	89.1	Midwife	9	11.5	69	88.5
University	19	8.1	215	91.9	Nurse/NP	2	28.6	5	71.4
Postgraduate degree	11	8.0	126	92.0					
**Employment Characteristics**					HCP discussed vacc. (*n* = 329)	27	10.5	231	89.5
Essential ^1^ worker (*n* = 564 ^2^)	38	10.2	335	89.8					
Could work from home (*n* = 563 ^2^)	31	10.9	254	89.1	COVID-19 vacc. (*n* = 561)	42	7.5	452	80.6
**Employment status (*n* = 560)**									
Worked full time/part-time/seasonal	29	8.5	314	91.5	**Worked outside home while pregnant (*n* = 564)**				
Work—maternity leave	15	9.1	149	90.9	Always/often	29	9.1	291	90.9
Unemployed/leaves of absence	8	15.1	45	84.9	Sometimes/rarely	5	5.2	91	94.8
					Never/did not work	18	12.2	130	87.8

^1^ Pandemic essential workers were employed in health/safety (first responders, healthcare workers), critical infrastructure operations, manufacturing, and supply chain sectors. ^2^ Not applicable responses excluded from analysis. ^3^ COVID-19 prenatal regional infection rates as in Table 2. Relationship between respondent age, household size, number of close contacts, and COVID-19 infection history evaluated by Pearson product–moment correlation. Mann–Whitney test used to determine the relationship between respondent education, income, and frequencies of work outside home while pregnant and limitations on trips outside home. Pearson Chi-Square (χ^2^) test used to assess relationships between respondent race, Indigeneity, immigrant status, gravidity, employment characteristics/status, domestic travel patterns, HCP vaccine discussion, and COVID-19 vaccination. SD-standard deviation, HCP-healthcare provider, OB/GYN-obstetrician/gynecologist, GP-general practitioner, NP—nurse practitioner. Infect—COVID-19 infection, Hist—history of infection, Vacc—vaccination.

**Table 6:** Logistic regression analysis of variables associated with history of COVID-19 infection.

Variable	COR	95% CI	AOR	95% CI
Gravidity (multigravid ^1^)	0.49	0.28–0.88	0.68	0.34–1.38
Household size	1.40	1.08–1.81	1.26	0.92–1.73
Racialized status (no ^1^)	2.71	1.18–6.24	2.78 *	1.19–6.50

^1^ Reference. COR—crude odds ratio, AOR—adjusted odds ratio, CI—confidence interval. * *p* < 0.05.

## Data Availability

The data that supports the findings of this study are available from the corresponding author (KPP) upon reasonable request. Summary data tables will be available at http://dx.doi.org/10.20381/ruor-29283 (accessed 12 March 2025) pending embargo.
